# The effectiveness of therapeutic eating disorder groups at Isis

**DOI:** 10.1186/2050-2974-3-S1-O6

**Published:** 2015-11-23

**Authors:** Rachel Signorini, Jeanie Sheffield, Isa Pfluger, Amanda Dearden

**Affiliations:** 1 The University of Queensland, St Lucia, QLD, Australia; 2 Isis - The Eating Issues Centre, Highgate Hill, QLD, Australia

## 

Isis – The Eating Issues Centre (Isis) offers empowerment-based therapeutic group work programs for adults with eating issues within the community. The current study evaluated the 18- and 10- Week Group programs provided by Isis using a mixed-method design. The pre and post-treatment data of 25 clients who participated in the 18- Week Group between 2010 and 2013, and 33 clients who participated in the 10- Week Group between 2009 and 2013 were analysed. Semi-structured interviews were also conducted with five clients who had completed the 18-Week Group, and six clients who had completed the 10- Week Group. Findings from the quantitative analyses revealed that participants of both programs experienced statistically and clinically significant improvements on the drive for thinness scale of the Eating Disorder Inventory-3 (EDI-3; Garner, 2004), as well as statistically (although not clinically) significant improvements on the bulimia, body dissatisfaction, interpersonal alienation, and interoceptive deficits scales. Qualitative findings revealed that participants were highly satisfied with the programs, identified numerous positive aspects of group work, and reported various beneficial outcomes. Collectively, the findings indicate that the 18- and 10- Week Group programs are helpful interventions for women with eating issues when accessed alongside individual support.

